# Prevalence of depressive symptoms and its related factors among students at Tra Vinh University, Vietnam in 2018

**DOI:** 10.3934/publichealth.2019.3.307

**Published:** 2019-08-22

**Authors:** Nguyen Thi Hong Tuyen, Truong Quang Dat, Huynh Thi Hong Nhung

**Affiliations:** 1 Department of Public Health, Tra Vinh University, Tra Vinh City, Vietnam; 2 Department of Public Health, Binh Dinh Medical College, Quy Nhon City, Vietnam

**Keywords:** depression, university students, Tra Vinh, Vietnam

## Abstract

**Objective:**

The study was conducted to estimate the prevalence of depressive symptoms and its related factors among students at Tra Vinh University, Vietnam.

**Methods:**

The instrument used was a questionnaire of socioeconomic-demographic characteristics, educational characteristics, and the self-reported depression scale collected from the Center for Epidemiological Studies-Depression (CES-D) originally published by Radloff in 1977. Scores of 16–21 were considered as mild to moderate symptoms of depression, and scores above 21 were considered as symptoms of major depression. The Chi-square test was performed to compare prevalences.

**Results:**

405 students (with 136 males and 269 females) aged 19 to 27 (the average age was 20.2) were interviewed. The mean score for the CES-D was 15.98, and the overall prevalence of depressive symptoms was 52.3%, including the mild to moderate symptoms of depression (24.2%) and the major depression (20.7%). The prevalence occupied 72.2% among students in poor and near-poor households (aOR = 3.06, 95% CI = 1.38–6.76, *p* = 0.006). The prevalence also was higher among those who had been drinking alcohol (59.7% with aOR = 2.02, 95% CI = 1.15–3.53, *p* = 0.014).). Depressive symptoms among first-year students were 39.9%, and 2nd-year students were 42.5% and tended to increase to 4th-year students (47.1%) with the *p*-value of 0.019.

**Conclusion:**

The overall prevalence of depression is relatively high among university students at Tra Vinh University, Vietnam. The prevalencesignificantly associates with characteristics such as household economics, behaviours and number of years studying at university. These results suggest that more attention should be directed to activities to reduce the prevalence of depressive symptoms, especially among students in the final years.

## 1. Introduction

Depression is a common mental disorder, characterized by persistent sadness and a loss of interest in activities that one usually enjoys, accompanied by an inability to carry out daily activities, for at least two weeks [1]. Also, people with depression often have the following manifestations: a loss of energy; a change in appetite; sleeping more or less; anxiety; reduced concentration; indecisiveness; restlessness; feelings of worthlessness; guilt, or hopelessness and thoughts of self-harm or suicide [1]. The symptoms of depression start at an early stage. They either remain persistent or increase at the alarming state, depending on the exposure to the environment and the potential capacity throughout the life of an individual [2].

Globally, the total number of people with depression was estimated to exceed 300 million in 2015 [3]. The global depression prevalence is estimated to be 4.4%. In Vietnam, it accounts for 4% of the nation's population. Depression occurs in every age and every country [4]. Depression is ranked by the World Health Organization (WHO) as the single most significant contributor to global disability (7.5% of all years lived with disability in 2015). Depression is also the major contributor to suicide deaths, which went up about 800,000 for each year [4].

There is evidence to suggest that university students are at higher risk of depression, despite being a socially advantaged population, but the reported prevalences have shown wide variability across settings. The prevalence of depression among university students in countries around the world ranged from 10% to 85% with a weighted mean prevalence of 30.6% [5]. Some studies in Vietnam showed that the prevalence of depression among university students was from 20.2% to 39.6% [6]–[9]. There were several factors related to depression among students recorded as gender, age, financial difficulties, posttrauma of a romantic relationship, disagreement with parents, physical attack, illness, and unsatisfactory scholarship achievement [5],[6]. However, the relevant factors differ between studies.

Tra Vinh University is a training facility for high human resources to serve mainly the provinces of the Mekong Delta in Vietnam and other parts of Vietnam with the number of over 10,000 full-time students with various specialities, including over 40 undergraduate majors and over 20 Advanced Diploma programs [10]. University students study hard and practice diligently to advance and achieve professional qualifications after graduation. At the same time, the majority of students who live away from home are likely to experience difficulties in housing conditions, financial constraints, and complications from private life. All of this puts pressure on students not only physically but mentally. Furthermore, in Vietnam, particularly in the study area, there is a scarcity of information on depression among university students. Therefore, determining the prevalence of depression and some factors related to the prevalence among university students of Tra Vinh University is very necessary, and the results will help to set out solutions to help them have the best learning environment and spiritual life.

Thus, the present study was designed to identify the prevalence of depressive symptoms using the Center for Epidemiologic Studies Depression Scale and its related factors among full-time students of Tra Vinh University (Vietnam) in 2018.

## 2. Materials and methods

### 2.1. Study design

This cross-sectional survey was conducted at Tra Vinh University in 2018. The university currently has nine faculties: Agriculture, Fisheries, Engineering and Technology, Medicine, Pharmacy, Economics, Law, Languages, Basic Science, Pedagogy, Language, Culture, Khmer Art of the South, State Administration, Office Administration, Tourism. Students among those nine faculties of Tra Vinh University were included in the study except for part-time ones and students admitted for less than six months. Full-time students are those who receive full-time training at the university. Part-time students are those who work or study to improve their professional knowledge or want to study other majors other than the industry they are doing at the same time.

### 2.2. Sample size

The sample size was calculated using the following formula: $n={\text{Z}}_{1-\alpha /2}^{2}\frac{\text{p}(1-\text{p})}{{\text{d}}^{2}}$; Where: *n* is the smallest sample size to be achieved; p is the expected prevalence: 38.9% [9] ; d is the absolute error; $Z_{1-\alpha /2}$ is Z statistic for a confidence level of 95%. The final sample size (n = 405) was determined by adjusting for expected non-response (10%).

### 2.3. Sampling method

Proportional stratified random sampling was employed to determine the number of students required for the study, per faculty. Simple random sampling was then used to select the students to whom the questionnaires were administered until the desired sample size was achieved.

### 2.4. Data collection

The questionnaire was randomly distributed to students on the day of the survey. One of the researchers explained the research objective briefly, and the second one distributed the hard copies of the questionnaires to the students. Verbal consent was taken before the research, and participation was voluntary. Students present on the day of the interview were included in the study (excluding part-time ones and students enrolled for less than six months), and filled questionnaires were collected on the same day.

The questionnaire mainly consisted of three parts:

1) The first part of the questionnaire comprised questions related to age, sex, ethnicity, religion, part-time jobs, marital status, household economy, behaviours.

2) The second part of the questionnaire consisted of questions related to educational characteristics such as faculties, year of study, academic results of the previous semester, and worry about careers after graduation.

3) The third part of the questionnaire contained questions from the self-reported depression scale obtained from the Center for Epidemiological Studies-Depression (CES-D), initially published by Radloff in 1977 [11], and it was translated into Vietnamese [6]. Currently, we have not found the CES-D toolkit in the Vietnamese language has been previously validated. The CES-D contains a 20-item measure that asks participants to rate how often they experienced symptoms associated with depression, such as restless sleep, poor appetite, and feeling lonely over the past week. Cronbach alpha for the scale in the current study was 0.8837. Response options range from 0 to 3 for each question (0 = rarely or not at all, 1 = some or little of time, 2 = occasionally or a moderate amount of time, and 3 = nearly every day for two weeks or 5–7 days). The score ranges from 0 to 60, with higher scores indicating greater levels of depression. Scores of 16–21 were considered as mild to moderate symptoms of depression, and scores above 21 were considered as major depression [11]–[12].

#### 2.4.1. Other variables

Sociodemographic information includes sex, age, and ethnicity in which the ethnicity was divided into the group of Kinh people and other groups of ethnic minorities. Kinh people account for over 86% of the entire population, followed by Tay, Thai, Muong, Khmer, Nung, Mong, and Dao ethnic groups accounting collectively for 10% of the total population. The Kinh is mainly in Vietnam and has more advantages than other ethnic groups [13]. Additionally, the questionnaire collected information about students' other characteristics (behaviours such as drinking and smoking, and the income of their family members). A student was considered smoking when smoking an average of over 20 cigarettes/day. A student was considered an alcohol drinker when drinking an average of one bottle of beer of 330ml (or equivalent)/day or more. To classify the household economy, we used the criteria specified in Decision 59/2015/Decision-P.M. of the Prime Minister of Vietnam [14].

### 2.5. Data processing

After detecting and correcting (or removing) corrupt or inaccurate records from a record set, data were entered into Epi Data 3.1 software and transferred to Stata 10.0 software for data analysis. Descriptive statistics (mean, percentage, and frequencies) were calculated to assess the percentage and levels of depression in the study participants. The Chi-square test for the difference in prevalence between two groups and a t-test for the difference in the mean score between male and female were utilized. The Chi-square test for trend was used to assess the trend of the prevalence of depressive symptoms by year of study, and academic results of the previous semester. Then, the multivariable logistic regression model was used to assess the relationship between different factors and the prevalence of depression. The independent variables from the Chi-square test with the *p*-value < 0.5 were included in the model. The reported results are adjusted OR (aOR) and 95% confidence intervals (95% CI) [15]. The *p*-value < 0.05 was considered significant.

### 2.6. Ethical considerations

The study was approved by the scientific council of Tra Vinh University. All students voluntarily participated. The data collected were kept confidential. The study results were to disseminated to the board of directors of Tra Vinh University in order to inform policies and interventions to improve the mental health of university students and paved the way for future studies.

## 3. Results

The data set included the records of 405 students (with 136 males and 269 females) aged 19 to 27 (The average age was 20.2), among which the Kinh was 69.9%, and other ethnic minority groups were 30.1%. All selected students participated in the study, giving a response rate of 100%.

Table 1 presented the numbers, means, and ranges of the CES-D by sex and age. The mean score for the CES-D for all students was 15.98 (mean = 15.72 for males; mean = 16.11 for females). There was no difference in the average score between men and women and by age group.

**Figure 1.**
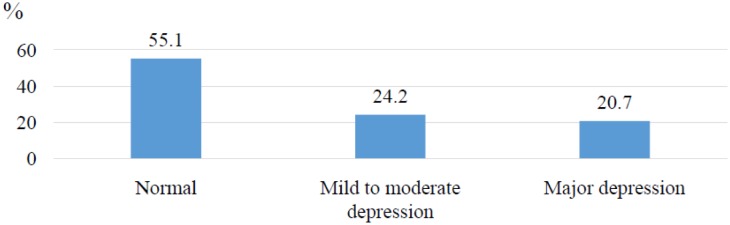
The percentage of depressive symptoms among students at Tra Vinh University, Vietnam (n = 405).

As shown in Figure 1, the prevalence of depressive symptoms was less than 50% in which mild to moderate level was 24.2%, and major depression was 20.7%. Table 2 showed the prevalence of depression according to age, sex, ethnicity, religion, part-time jobs, marital status, and household economy. The prevalence of depression among males was 46.3%, among females was 44.2%. But this difference was not significant (*p* = 0.69). Among these independent variables, only the household economy was related to the prevalence. The general prevalence among students being in poor and near-poor households was 72.2%, much higher than that among the other students *(p* = 0.001).

The overall prevalence of depression among students in their lifestyles was presented in Table 3. It indicated that the independent effect of alcohol consumption on the prevalence was significant. Namely, the prevalence among students with alcohol consumption was 59.7%, higher than that among the other students (the *p-*value of 0.005). Smoking was not associated with the prevalence of depression in the data set.

Table 4 showed the prevalence of depressive symptoms among students according to their educational characteristics. The general prevalence of depression tended to increase with the number of years of students studying at university. The prevalence among the first-year students was 39.9%, but among the fourth-year ones went up 47.1% (*p* = 0.019). Depression was not associated with students' worry about future careers (*p* = 0.051).

Table 5 presented the results obtained from the multivariable logistic regression analysis. Variables including household economy, alcohol consumption, year of study, and worry about future careers after graduation were significant independent variables in predicting the prevalence of depressive symptoms among students. Students being in poor and near-poor households were more likely to experience depression than the other group (aOR = 3.06, 95% CI = 1.38–6.76, *p* = 0.006). Students with alcohol consumption were more likely to experience depression than the other group (aOR = 2.02, 95% CI = 1.15–3.53, *p* = 0.014). The third-year students were more likely to experience depression than the first-year students (aOR = 1.84, 95% CI = 1.11–3.06, *p* = 0.019). Students who were worried about their careers after graduation were more likely to experience depression than the other group (aOR = 2.41, 95% CI = 1.11–5.26, *p* = 0.027).

**Table 1.** Numbers, mean scores of the CES-D scores according to sex and age at Tra Vinh University, Vietnam.

Age	Total		Males		Females		*p*
	No	Mean	No	Mean	No	Mean	
19	176	15.46	70	15.94	106	15.14	0.500
20	76	14.62	18	14.44	58	14.67	0.895
21	96	17.58	22	16.05	74	18.04	0.350
> 21	57	16.70	26	15.73	31	17.52	0.434
Total	405	15.98	136	15.72	269	16.11	0.638

**Table 2.** The overall prevalence of depressive symptoms among students at Tra Vinh University, Vietnam according to their demographic characteristics (n = 405).

Variables	Values	Total	Depressive symptoms		*p*
			Yes (%)	No (%)	
Age	19	176	74 (42.1)	102 (57.9)	0.638
	20	76	33 (43.4)	43 (56.6)	
	21	96	47 (49.0)	49 (51.0)	
	> 21	57	28 (49.1)	29 (50.9)	
Gender	Male	136	63 (46.3)	73 (53.7)	0.690
	Female	269	119 (44.2)	150 (55.8)	
Ethnicity	Kinh	283	126 (44.5)	157 (55.5)	0.798
	Others	122	56 (45.9)	66 (54.1)	
Religion	Yes	147	72 (49.0)	75 (51.0)	0.217
	No	258	110 (42.6)	148 (57.4)	
Part-time jobs	Yes	108	52 (48.2)	56 (51.8)	0.434
	No	297	130 (43.8)	167 (56.2)	
Marital status	Without a partner	265	120 (45.3)	145 (54.7)	0.848
	With a partner	140	62 (44.3)	78 (55.7)	
Household economy	Poor and near-poor	36	26 (72.2)	10 (27.8)	0.001
	Other	369	156 (42.3)	213 (57.7)	

**Table 3.** The overall prevalence of depressive symptoms among students at Tra Vinh University, Vietnam according to their lifestyles (n = 405).

Variables	Values	Total	Depressive symptoms		*p*
			Yes (%)	No (%)	
Smoking	Yes	4	2 (50.0)	2 (50.0)	0.838
	No	401	180 (44.9)	221 (55.1)	
Alcohol consumption	Yes	72	43 (59.7)	29 (40.3)	0.005
	No	333	139 (41.7)	194 (58.3)	

**Table 4.** The overall prevalence of depressive symptoms among students at Tra Vinh University, Vietnam according to their educational characteristics (n = 405).

Variables	Values	Total	Depressive symptoms		*p*
			Yes (%)	No (%)	
Faculty	Health	75	29 (38.7)	46 (61.3)	0.226
	Others	330	153 (46.4)	177 (53.6)	
Year of study	1	203	81 (39.9)	122 (60.1)	0.019*
	2	73	31 (42.5)	42 (57.5)	
	3	112	62 (55.4)	50 (44.6)	
	4	17	8 (47.1)	9 (52.9)	
Academic results of the previous semester	Good and excellent	76	37 (48.7)	39 (51.3)	0.242*
	Average	305	137 (44.9)	168 (55.1)	
	Weak	24	8 (33.3)	16 (66.7)	
Worry about future careers	Yes	368	171 (46.5)	197 (53.5)	0.051
	No	37	11 (29.7)	26 (70.3)	

Note: The p (*) is the probability value obtained in the Chi-square test for trends of the prevalence of depression across the year of study and academic performances.

## 4. Discussion

There are many tools used to assess depression levels in research populations [11]–[18]; even the same tool set used, but the cut-off points are chosen differently in various studies [11],[19]. In the present study, we utilized the CES-D scale, a self-report depression scale, that has been widely used in population surveys across the world and has satisfactory levels of reliability and validity in numerous cultures [20]–[21]. The results revealed that the mean CES-D score was 15.98 and 52.3% of students at Tra Vinh University exhibited depressive symptoms (CES-D scores of 16 or above). The mean CES-D score in our study was slightly higher than the mean CES-D score of 14.58 in a study on an ethnographically diverse sample consisting primarily of Asian Americans, European Americans, Native Hawaiians, and Pacific Islanders [21]. The figures clearly showed that education managers could not assume that their university students are free from mental health problems and suggest that screening and early intervention for problems such as depression might be an appropriate course of action.

**Table 5.** The results obtained from the multivariable logistic regression analysis for the overall prevalence of depressive symptoms among students at Tra Vinh University, Vietnam.

Variables	Values	aOR (95% CI)	*p*
Religion	Yes	1.22 (0.80–1.88)	0.355
	No	Reference	
Part-time jobs	Yes	1.09 (0.68–1.74)	0.728
	No	Reference	
Household economy	Poor and near-poor	3.06 (1.38–6.76)	0.006
	Other	Reference	
Alcohol consumption	Yes	2.02 (1.15–3.53)	0.014
	No	Reference	
Faculty	Health	0.68 (0.39–1.19)	0.175
	Others	Reference	
Year of study	1	Reference	
	2	1.08 (0.60–1.94)	0.799
	3	1.84 (1.11–3.06)	0.019
	4	1.16 (0.40–3.38)	0.780
Academic results of the previous semester	Good and excellent	Reference	
	Average	0.71 (0.42–1.21)	0.206
	Weak	0.45 (0.16–1.29)	0.137
Worry about future careers	Yes	2.41 (1.11–5.26)	0.027
	No	Reference	

Note: Summary of the model: Number of obs = 405, LR chi2 (11) = 33.97, Prob > chi2 = 0.0004, Log likelihood = −261.66019, Pseudo R2 = 0.0610.

This overall prevalence of depressive symptoms is in the range of the prevalence of depression among students, consistent with other studies 5 [5], but much higher than the prevalence reported by some studies in Vietnam [6]–[8]. For example, the prevalence of depression among second-year medical students at Hanoi Medical University was 28.46% but instruments used comprised of the Eysenck Personality Questionnaire (EPQ) and the Reynolds Adolescent Depression Scale (RADS) [7]. Another study carried out on second-year medical and dentistry students at the University of Medicine and Pharmacy in Ho Chi Minh City, Vietnam by using DASS—21 questionnaires showed the prevalence of depression was 22.4% [8]. In our study, with the cutoff of 22, the prevalence of depression was much lower than that found among medical students at the University of Medicine and Pharmacy in Ho Chi Minh city, Vietnam using the same cut-off point (CES-D scores of 22 or above) [9]. This difference may be due to the medical training program is more stressful than other training ones [10]. This difference may also be due to ethnic differences; up to 30.1% of Tra Vinh University's students belong to ethnic minority groups while most of the students at the University of Medicine and Pharmacy are Kinh people [9]. When observed with the cutoff of 16, the prevalence was close to that among students in Hai Phong, Vietnam (46.9%) [23] but much higher than that in Beijing (24.8%) [24] and a bit lower than in Cambodia (50.6%) [25], in Pakistan (60.4%) [26]. These studies also used the same cut-off point of 16 (CES-D scores of 16 or above). While a study in Nigeria recorded the prevalence of depression among university students was 58.2% measured by the Patient Health Questionnaire-9 (PHQ-9) [27]. Another survey among medical undergraduates in the Faculty of Medicine in Sri Lanka using the Beck Depression Inventory (BDI) revealed that the prevalence of depression 19% [28]. Possible explanations for the variation in the prevalence of depression found in this present study and previous studies conducted among university students are likely due to the change in the instruments used. Differences in the time of the study, as well as differences in culture and lifestyle across the regions where the studies were carried out, may account for variations in the final results. This difference may also be due to university training programs which vary between countries.

Several studies indicate that the prevalence of depression among university students is higher than that of other populations [5],[29]–[30], and this has been found in the present study. The learning pressure of students can partly explain this difference. Everyday life stress as well as education pressure increases the vulnerability of the students, and therefore, attributes in the high prevalence of psychological illnesses among students [31]. Many studies conducted on medical students also showed that this proportion is quite high due to the specificity of the medical speciality [32]–[33]. The prevalence of depressive symptoms among students studying health-related majors at Tra Vinh University was 38.7%, higher than that in Japanese employees [30]; however, it was not different from that among the other (38.7% and 46.4% with the *p*-value of 0.226). The reason for this is unclear, but there may be other factors related to the prevalence of depression rather than specialities that students are studying.

The present study found no significant statistical relationship between depression and demographic characteristics such as age, sex, ethnicity, religion, part-time jobs, marital status, and smoking behaviour. A possible explanation for this is that the traits did not predispose respondents to depression. This finding contradicts the findings from other studies. These studies showed that some demographic variables were risk factors for depression [12],[30]. For example, the research done in Japan showed that the prevalence of depression among Japanese Employees on the CES-D varied meaningfully by smoking behaviour [30]. This difference in the proportion of depression among participants according to gender in our study was not statistically significant, which is consistent with several previous studies [32],[34]. This finding might originate from the fact that female university students have a similar experience of the same pressure. However, other studies showed either a higher prevalence of depression in females [35]–[37] or males [38]–[39].

The present study showed that economic difficulties and alcohol consumption remain apparent risk factors of depressive symptoms. Students in economically disadvantaged families (near-poor and poor households) had a higher prevalence of depression, and the finding agrees with another study on the same theme [40]. Thus, we can deduce financial difficulties might be behind this finding, interfering with the student's mood. Therefore, household economy, year of study, and alcohol consumption may be highly useful variables that predict the prevalence of depressive symptoms on CES-D scores. Furthermore, it can be said that poor and near-poor households and alcohol consumption are quite popular in Vietnam [41]–[42].

In this study, the prevalence of depressive symptoms did not alter significantly by students' academic performance but varied considerably by other educationalfactors such as the year of study. Poor academic performance was found to be associated with depression in the reviewin Nigeria [43]. Our research revealed that the prevalence of students suffering from depressive symptoms tended to increase to the final years (*p* = 0.019). The results obtained from the multivariable logistic regression analysis showed that the third-year students were more likely to experience depression than the first-year students (aOR = 1.84, 95% CI = 1.11–3.06, *p* = 0.019). This percentage among first-year students and sophomores was 39.9% and 42.5%, respectively, and among fourth-year students was 47.1%. The findings in the current study are different from some other studies, which showed that first-year students accounted for the highest depression [26],[44] or no difference in the prevalence of depression according to years of training [34]. However, our findings are in line with previous studies; for instance, studies from Pakistan and Thailand reported a higher level of stress among third and fourth-year students [45]–[46]. The reasons for such high levels of depression among fourth-year students are involved and reflect both the environment and personal characteristics. In the last university years, there are significant changes in the students' lifestyle, such as preparing exams, worrying about jobs after graduation. These may lead to the development of depressive symptoms among students [47]. Finding a job after graduation is a significant concern for all students entering a university, and it is not always accessible in Vietnam in the current period [48]. The students who were worried about their careers after graduation were more likely to experience depression than the other group (aOR = 2.41, 95% CI = 1.11–5.26, *p* = 0.027). Thus, the prevalence of depression was higher among final year students can be partly explained by this reason.

### 4.1. Study limitations

This study has several limitations that might need attention. First, this study was confined to full-time students; therefore, any attempt to generalize this finding to all kinds of students of Tra Vinh University should be made with caution. Second, the sample size for each faculty was relatively small to test the real association between the factor and outcome variables; consequently, a larger sample for each faculty is required to verify the findings. Third, collected data was based on self-reporting; hence, under or over-reporting of behaviour might have affected the results; for this reason, it can be evaluated by further studies in depth by quantitative and qualitative methods. Fourth, currently, we have not found the CES-D toolkit in the Vietnamese language has been previously validated. Besides, the CESD is not accurate for assessing a clinical depression, which was another limitation in our study [49]. Last but not least, an additional limitation to this study is its cross-sectional design, in which it was impossible to define the temporal relationship between cause and consequence.

## 5. Conclusion

The overall prevalence of depressive symptoms is relatively high among university students at Tra Vinh University, Vietnam. The prevalence relates to characteristics such as household economy, behaviours, and the number of years studying at university. These results suggest that more attention should be directed to activities to reduce the prevalence of depression, especially among students in the final years.
